# A custom-built planar biaxial system for soft tissue material testing

**DOI:** 10.1016/j.ohx.2023.e00475

**Published:** 2023-09-19

**Authors:** Salvatore Pasta, Chiara Catalano, Fabrizio Crascì, Roberta Scuoppo

**Affiliations:** a Department of Engineering, Viale delle Scienze, Università degli Studi di Palermo, Palermo, Italy; b Department of Research, IRCCS-ISMETT, Palermo, Italy

**Keywords:** Biaxial system, Material testing, 3D printing, Biomechanics, Soft tissue

## Abstract

Accurate material characterization of soft tissues is crucial for understanding the physiopathology of cardiovascular diseases. However, commercial biaxial testing systems are expensive, prompting the need for affordable custom solutions. This study aimed to develop a low-cost custom biaxial system capable of accurately characterizing the mechanical behavior of soft tissues. The biaxial system was constructed using 3D printing technology and non-captive linear actuators for precise displacement control. A real-time marker tracking system was implemented to estimate dis-placements without the need for costly hardware. The system's performance was evaluated through tests on a calibration spring and frozen porcine aorta samples. The linear actuators demonstrated excellent response to user position input after motor tuning, showing no discrepancies between commands and actual positions. The experimental testing of the calibration spring showed good agreement with the analytical solution, validating the system's ability to accurately test materials. Testing on porcine aorta samples revealed stress–strain responses consistent with existing literature, accounting for potential variations due to tissue preservation and regional material property heterogeneity. Overall, this custom biaxial system demonstrates promising performance in accurately assessing the mechanical behavior of soft tissues, providing researchers with a valuable tool for cardiovascular disease research and tissue engineering applications.

## Graphical abstract

**Figure:**
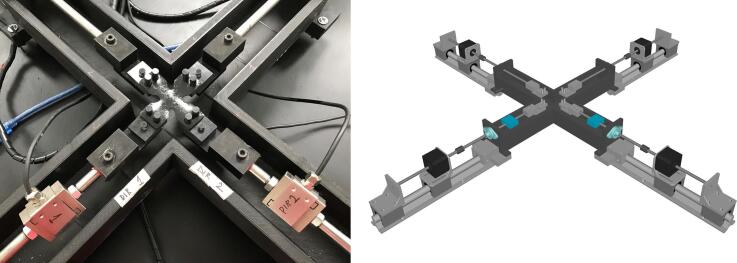

Specifications table

**Table:** 

Hardware name	BixTester
Subject area	• Engineering and materials science
Hardware type	• Other: Material Testing
Closest commercial analog	• TA Instruments: ElectroForce Planar Biaxial TestBench Instrument • CellScale BioTester
Open source license	CC-BY 4.0
Cost of hardware	€3230
Source file repository	https://doi.org/10.17632/ftzs4yrztd.1

## Hardware in context

The biomechanical characterization of tissue ex-vivo is paramount for assessing organ physiology, physiopathology, and developing constitutive relationships for in-silico computational models [1], [2], [3]. Several material testing approaches have been developed to estimate the ex-vivo material response under different loading conditions [4], [5], [6]. Testing modalities, such as tensile testing [7], planar biaxial testing [8], peeling testing [9], bulge inflation testing [10], indentation [11], and atomic force microscopy [12], have been employed for the assessment of the diseased human aorta. Additionally, shearing testing has been adopted for characterizing the complex anisotropic behavior of the left heart myocardium [13]. Experimental techniques, including digital image correlation [14], fluorescence microscopy [15], and multiphoton imaging [16], complement the material testing by providing a detailed analysis of the strain field in the investigated material. These tests allow for characterizing not only the material response un-der physiological deformation but also the failure behavior of human or animal tissues, enabling the development of novel damage criteria.

However, the commercial devices available in the market are often prohibitively expensive, costing more than €50,000, and their prices increase based on the required testing modality (displacement- versus force-controlled testing), gripping mechanisms, and implemented technological solutions (e.g., hardware and software). Additionally, if experimental techniques are needed, they further contribute to the overall system cost. Customizing a commercial system for a specific testing setup can be problematic. For in-stance, in the case of planar biaxial testing, most systems have a limited displacement range that does not allow for material rupture. Consequently, there is a pressing need to develop inexpensive and open-source planar biaxial material testing systems that enable accurate estimations of biomechanical properties in human and animal tissues. Such systems should also be easily customizable by researchers.

This study presents the design and development process of an affordable, 3D-printed planar biaxial testing system (namely, the BixTester) that enables highly accurate and precise mate-rial testing of soft tissues. The proposed technological solution incorporates standard features found in many commercial devices, including real-time acquisition of the sample displacement field and automated grip motion, all accessible through a user-friendly graphical interface. The planar biaxial system is equipped with a temperature-controlled bath and offers a large travel range (180 mm along one axis direction) for material testing under displacement control. The performance of the proposed biaxial system was evaluated with several tests and by comparing the material response of a spring with given analytical solution. Additionally, biaxial testing was conducted on porcine aorta, and the results are presented and discussed.

## Hardware description

**Fig. 1:**
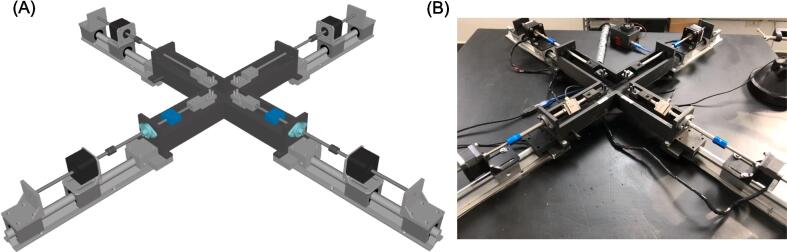
(A) CAD model of biaxial system assembly and (B) actual manufactured planar biaxial system.

The custom-built planar biaxial system consists of the following components: i) the testing platform with four linear actuators, ii) the specimen testing chamber, iii) the load cells, iv) the electronic acquisition board, v) the gripping mechanism, and vi) the graphical user interface (GUI) program for control and data acquisition (Fig. 1).

Similar to commercial systems, this testing machine is developed with high precision and accuracy of motion to capture the response of hyperplastic soft tissues under low force values, both in dry and immersed conditions. The system is designed to be compact and easily customizable to meet specific user requirements. The key aspects implemented in the system are as follows:

• low-cost system (approximately <€3,500) achieved through the utilization of 3D printed parts and simple design specifications, enabling user customization and compactness;
• real-time acquisition of displacements using marker tracking and force measurements during material testing;
• fully-automated system with configurable parameters to achieve high-precision motions under large displacement values.

### Testing platform and actuators

**Fig. 2:**
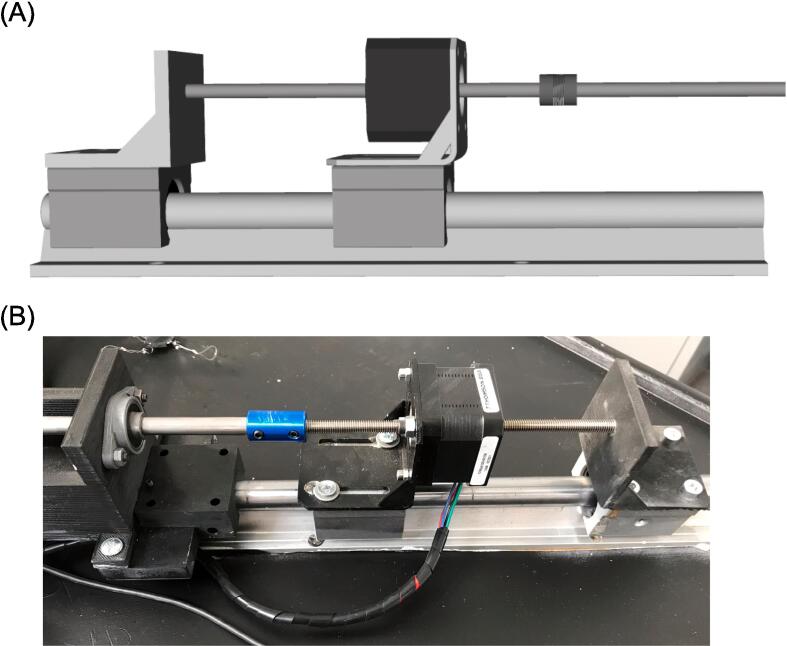
(A) CAD model of linear actuator and (B) actual linear actuator showing the non-captive linear motor mounted on the slider.

The geometry of the testing platform was developed using the computer-aided design software, RhinoCeros (v.7, Robert McNeel & Associates, USA). The overall configuration was derived from current commercial systems and guided by our experience in soft tissue material testing. The design of the linear actuators was crafted in alignment with established machine design principles. The testing platform is configured in a cruciform shape, allowing material testing along two orthogonal and planar directions. The sample is positioned at the midpoint of the cruciform platform to be simultaneously stretched by four linear actuators during material testing. Each linear actuator is constructed using non-captive lead-screw step motors (MLN Nema 17 A10, Thomson Linear Motion, UK), which provide linear motion by utilizing a rotating lead screw as the rotor (Fig. 2). When the current is applied to the stator coils, the magnetic field is generated, causing the lead screw to move linearly along the motor axis while being secured against rotations. To achieve this, the distal end of the lead screw is connected to an external carrier, which prevents lead rotation but allows translation along the motor axis. Both the linear step motor and carrier are mounted on two linear sliders (SBR16UU) assembled on a rail (SBR16). While the slider with the step motor remains fixed to the rail, the carrier can translate along the rail direction allowing translation of the grips. This configuration results in a compact design, with the step motor measuring 42x42x31 mm, a stroke of 100 mm (equal to the lead screw length), and a maximum axial load capacity of 224.4 N.

### Specimen testing chamber

The specimen testing chamber is designed in a cross-shaped configuration with a length of 380 mm and a width of 60 mm, providing an effective displacement range of 180 mm along each axis. The chamber offers an effective testing area of 44x44 mm, which is sufficient for testing specimens from the heart or great vessels. To minimize fluid usage, the chamber has a height of 30 mm. The fluid is heated by a Peltier ceramic plate placed inside the chamber and controlled using a W1209 control board. The bed of the testing chamber is slightly inclined towards the flow outlet, facilitating fluid drainage after the testing session.

Owing to the sizable dimensions (380 mm), outsourcing was a practical solution for fabricating the testing chamber. Specifically, the CraftCloud web platform was instrumental in identifying a suitable manufacturer. Subsequently, the specimen testing chamber was expertly crafted from PLA material using Fused Deposition Modeling (FDM) 3D printing technology. To ensure water resistance, a silicone layer was applied to the inner surface of the testing chamber.

### Specimen gripping system

**Fig. 3:**
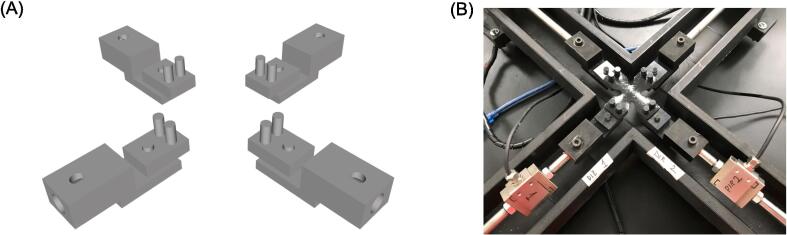
(A) CAD models of specimen grips and (B) examples of rubber-like specimens mounted on the grip mechanisms.

Four custom-made grips were designed in two distinct parts to facilitate the mounting of soft tissue samples and enable easy exchange with other gripping mechanisms (Fig. 3). The first part is utilized to secure each grip to the stainless steel shaft (8 mm in diameter) connected to the linear actuator using rigid couplings. Grooves generated on the specimen testing chamber house several O-rings, which can be employed to seal the motor shaft. To ensure wobble-free translation as the carrier moves along the rail, each shaft is aligned with the motor axis using four axial bearings fixed to the specimen testing chamber using screws.

The second part of the gripping system consists of the connector with the specimen. This can be accomplished by balanced pulley sample mounting grips, allowing specimen mounting using suture hooks, as is commonly done in other biaxial systems. This connector also presents several hole to allow the connection with steel racks for specimen mounting. To ensure proper immersion of the sample during testing and prevent corrosion of load cells, the specimen gripping system is positioned 12 mm below the level of the specimen testing platform. The specimen gripping system is manufactured using stereolithography (SLA) rapid prototyping technology, specifically utilizing a rigid black-colored resin (Form 3B+, Formlabs, USA).

### Force sensing

Two miniature load cells (25x30x12 mm) are equipped on the arms of the biaxial testing system to measure the load in the X-Y plane directions (refer to Fig. 3B). Based on our experience with biaxial testing of the human aorta, the load cells (DYLY-106, Calt Sensor, CN) have a capacity of 30 N and a sensitivity of 1.0 mV/v with non-linearity and repeatability of 0.03% of full scale. As the load cells are constructed from a steel alloy, they are mounted in line with the motor axis but positioned higher than the specimen gripping system to prevent corrosion in case of contact with the chamber fluid. The load cells have a 6 mm thread depth, allowing direct assembly to the ends of the shafts (i.e., the motor and grip ends).

Each load cell cable is connected to an analogical signal conditioner (JY-S60 Series, Calt Sensor, China). This enables the conversion of the Wheatstone bridge signal output of the load cell into a 4–20 mA amplified current signal, with an accuracy of over 2% of full scale. The signals from the load cells are acquired using a C Series DAQ Ethernet chassis (CDAQ-9181, National Instruments, USA), which provides 8 analog current-input channels and a 200 kS/s sample rate (NI-9203, National Instruments, USA) for real-time acquisition and visual analysis through the LabView interface. Both the load cell conditioners and the DAQ chassis are powered with 12 V and 5 V DC current, respectively.

### Real-time displacement acquisition

A webcam (C910, Logitech, Switzerland) positioned perpendicular to the testing chamber is used to continuously capture images of the specimen sample's field of view during stretching. A semi-rigid arm enables the camera to be positioned close to the sample area, allowing for marker tracking throughout the entire duration of the test. The camera is connected to the LabView software using a USB port. Local displacements along orthogonal loading directions are measured using four acrylic-painted markers on the sample surface.

**Fig. 4:**
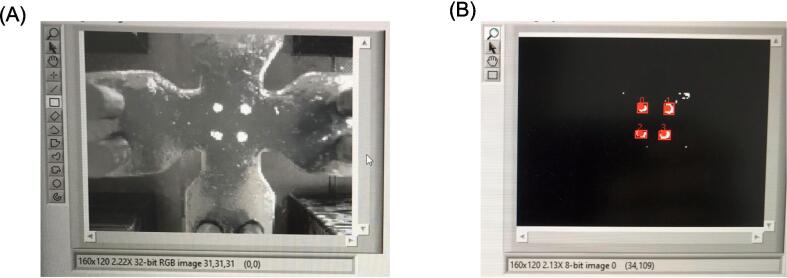
**(A)** Picture of specimen with white markers and (B) tracked marker positions as a result of LabView script.

Using the LabView Vision Development Module, a block diagram is developed to track the centroid of the markers by pixel-weighted operation through pattern matching of the acquired images. To facilitate real-time acquisition and analysis of the markers, the images are scaled to a resolution of 380×240 pixels at a frame rate of 30 fps, and then transformed into monochrome images. The LabView program's front panel offers tools to enhance contrast and brightness, adjust settings for filling potential holes within the markers and specify the minimum or maximum pixel size of objects to be tracked during testing. Additionally, an image crop of the sample can be generated to exclude the region of suture hooks. The program allows for the tracking of both black and white markers, ensuring clear marker shapes with stable centroids for accurate measurements during material testing (Fig. 4). Furthermore, the LabView program provides a grabbing tool to capture an image of the sample along with a ruler, enabling the measurement of specimen dimensions in millimeters.

### Graphical user interface

**Fig. 5:**
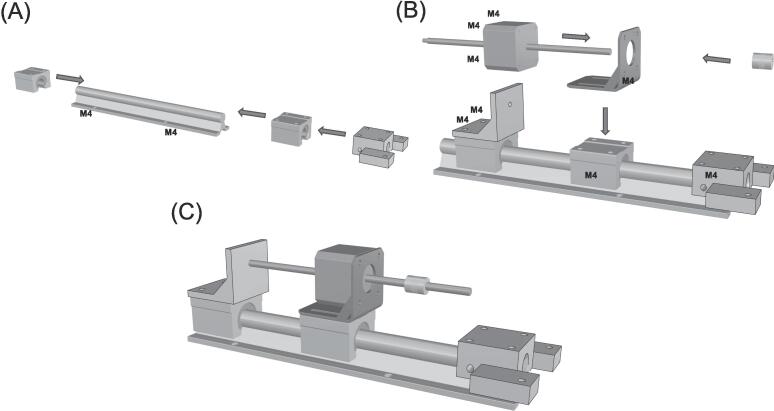
(A) Fasten the rails with M4 screws and then insert the sliders and the platform support; (B) Fasten the motor slider, carrier and platform support and then mount the motor with its support and the joint; (C) final assembly of the linear actuator.

The GUI program developed in LabView not only facilitates real-time displacement analysis but also allows for control of the biaxial system, similar to commercial devices (see Fig. 5). Through the GUI program, users can command the linear actuators to set up the grip positions and velocities in the plane by communicating with the driver control board. To control the motors, the program utilizes the machine control toolkit library, which enables the transmission of G-code commands to each motor via VI inputs. The G-code comprises simple block commands specifying coordinates in the X-Y plane, sent to the motors as string inputs when connected to the computer via USB. The machine control toolkit library is based on the open-source GrBl firmware, commonly used in milling machines, and is implemented on an Arduino board (R1 Arduino board, Arduino, Italy) with the CNC shield V3.0. This setup allows for the management of two DRV8825 stepper drivers. To achieve higher resolution, the motors aligned along the same direction are connected in series and then linked to the stepper driver. The DRV8825 supports 1/32 microstepping, resulting in a displacement resolution of 31 μm for each motor. To maintain optimal performance, a 30x30 mm fan is employed to cool the drivers, with the driver control board housed in a 3D-printed enclosure and powered by a 24 V supply.

### Validation and characterization

The performance of the biaxial system is assessed through various tests and experiments conducted on springs and porcine biaxial samples. The accuracy of each linear actuator's motion is evaluated by comparing the target positions set in the GUI program with the displacement measurements obtained using a digital caliper. Three tests are performed for each motor, and the differences between the input and measured values are analyzed. This information is used to optimize the Grbl firmware settings by adjusting the step per mm for each linear actuator. The camera-based marker tracking tool is validated by acquiring and analyzing images of a grid composed of black dots with a diameter of 2.3 mm and a pitch of 7 mm. The LabView program is used to track and analyze the positions of these markers.

To validate the response of the biaxial system, a stainless steel spring with certified mechanical properties is utilized. The minimum reaction load and the linear relationship between force and displacement in the spring are tested. The spring has an initial tension of 1.39 N, which represents the minimum force required to separate all its coils. By using the spring constant of 0.23 N/mm, the expected force can be estimated within the range of the actual displacements recorded during the uniaxial experimental testing of the spring. The results from testing the spring in both planar directions are then compared to the analytical solution as obtained from the product of spring constant and the displacement value.

Finally, biaxial tests are conducted on several frozen porcine aorta using a previously published protocol to further evaluate and validate the performance of the biaxial system [17], [18], [19]. In brief, square porcine samples (10x10 mm) were cur from the animal tissues and then steel racks are used for mounting the sample on the plan biaxial system. Each test, the sample was pre-conditioned at 7% strain rate for ten cycles. Force and displacement were obtained to compute the engineering stress and strain.

## Design files summary

**Table:** 

**Design file name**	**File type**	**Open source license**	**Location of the file**
Assembly	CAD	CC-BY-4.0	https://doi.org/10.17632/ftzs4yrztd.1
Testing Platform	CAD	CC-BY-4.0	https://doi.org/10.17632/ftzs4yrztd.1
Motor Support	CAD	CC-BY-4.0	https://doi.org/10.17632/ftzs4yrztd.1
Grip Base	CAD	CC-BY-4.0	https://doi.org/10.17632/ftzs4yrztd.1
Grip	CAD	CC-BY-4.0	https://doi.org/10.17632/ftzs4yrztd.1
Carrier	CAD	CC-BY-4.0	https://doi.org/10.17632/ftzs4yrztd.1
Platform Support	CAD	CC-BY-4.0	https://doi.org/10.17632/ftzs4yrztd.1
Control Board Case	CAD	CC-BY-4.0	https://doi.org/10.17632/ftzs4yrztd.1
Biaxial Control Final	Software	CC-BY-4.0	https://doi.org/10.17632/ftzs4yrztd.1

Assembly: CAD model of all assembled parts as shown in Fig. 1.

Testing Platform: STL file for 3D printing of the testing platform; fabrication was done out of sourcing due to the large design of the platform.

Motor support: STL file for 3D printing of the support to hold the motor. This can be printed with PLA and layer thickness of 0.1 mm with 60% infill and not any material support.

Grip Base: STL file for manufacturing the part of the grip to be connected with the steel shaft. Rigid resin is recommended for 3D printing.

Grip: STL file of the gripping mechanism using suture hooks for specimen mounting, Rigid resin is recommended for 3D printing.

Carrier: STL file of the support to constraint motor rotation and allows shaft motion. This can be printed with PLA and layer thickness of 0.1 mm with 60% infill and not any material support.

Platform Support: STL file of the part connecting the metallic slider to the platform support. This can be printed with PLA and layer thickness of 0.1 mm with 60% infill and not any material support.

Control Board Case: STL file of the case for both the Arduino board and CNC shield. This can be printed with PLA and layer thickness of 0.1 mm with 60% infill (no support).

Biaxial Control Final: Labview application to control the biaxial system. The application requires the Machine Control Toolkit, which can be downloaded among the material source.

## Bill of materials summary

**Table:** 

**Designator**	**Component**	**Number**	**Unit Cost (euro)**	**Total Cost** **(euro)**	**Source**	**Material**
Platform		1	35.4	35.4	Craftcloud	PLA
Rail −40 cm	SBR16	4	15.7	62.8	RepRapWorld	Steel
Linear Slider	SBR16UU	8	6.5	52.0	RepRapWorld	Steel
Motor Support	NEMA17MNTBRC	4	7.2	28.8	RepRapWorld	Steel
Bearing	KFL8	4	3.4	13.6	RepRapWorld	Steel
Flexible Joint	FLEXCOUPLING8M8	4	4.2	16.8	RepRapWorld	Steel
Rods 8 mm	M8SMOOTH	4	6.2	24.8	RepRapWorld	Steel
Carrier		4	4.1	16.4	Craftcloud	PLA
Platform Support		4	8.8	35.2	Craftcloud	PLA
Base Grip		4	1.4	5.6	Craftcloud	PLA
Grips		4	1.2	4.8	Craftcloud	PLA
Board Case		1	3.5	3.5	Craftcloud	PLA
Nema Motor	MLN17A10-M06010P15000N	4	233.7	934.8	Thomson Linear	Electronics
Motor Cable	B089RC7QPQ	1	9.0	9.0	Amazon	Electronics
CNC Shield	B07CZDC9TZ	1	10.0	10.0	Amazon	Electronics
Driver	DRV8825	2	8.9	17.8	Amazon	Electronics
R1 Board	IT-EL-CB-001	1	15.9	15.9	Amazon	Electronics
EndStop	B08R5NZDMH	4	2.5	10.0	Amazon	Electronics
Power Supply	ALIM30A	2	12.6	25.2	Amazon	Electronics
Camera	960–001055	1	73.0	73.0	Amazon	Electronics
Camera Holder	B07CYPF8RD	1	24.9	24.9	Amazon	Metal
Load Cell	DYLY-106	2	32.4	64.8	CaltSensor	Electronics
Amplifier	JY-S60	2	11.5	23.0	CaltSensor	Electronics
NI Module	NI-9203	1	1025.0	1025.0	National Instruments	Electronics
NI Chassis	CDAQ-9181	1	685.0	685.0	National Instruments	Electronics
Temperature Board	W1209	1	11.0	11.0	Amazon	Electronics
Peltier Plate	TEC1-12703	1	1.5	1.5	Amazon	Electronics

## Build instructions

### Testing platform and actuators

The biaxial system can be assembled onto any rigid table using several standard metric screws (M4 and M6) as shown by Fig. 5. The design of the biaxial system is simple, making assembly easy and requiring no expertise. In brief, the four rails (SBR16) are aligned in a cruciform configuration and securely mounted to a rigid metallic desk using M6 screws. Once the rails are fastened, the eight linear sliders (four for the motors and four for the carriers) can be easily assembled to mount the motor supports and 3D-printed carriers using M4 screws. The four linear sliders (SBR16UU) with the linear motors are attached to the rails using 2 mm trapezoidal screws. This ensures the translation of the specimen grips during motor motion, while allowing for the release of the trapezoidal screws to optimize the motor stroke and translation of the entire linear actuator system. The motor lead screws are connected to the steel rods of the gripping mechanism using four rigid couplings with a bore diameter of 8 mm. The rods are then aligned to the testing platform using four axial bearings (KFL8) fixed to the testing platform frame.

### Specimen testing chamber

**Fig. 6:**
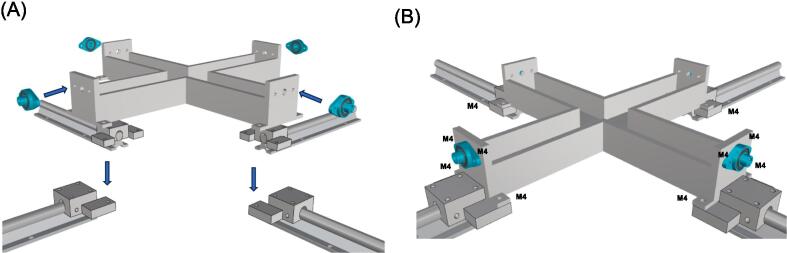
(A) Mount the specimen testing chamber on the four platform support and then mount the linear joints to the testing chamber; (B) Fasten the testing chamber and the linear joint with M4 screws.

The specimen testing chamber is part of the testing platform and therefore does not require any specific building instructions (Fig. 6). The testing chamber is connected to four rails (SBR16) through a custom-made support as represented by the platform support (see the bill of materials). This support is designed to prevent direct contact between the testing chamber and the rigid metallic desk. The fluid within the chamber is heated using a Peltier ceramic (TEC1-12706) to maintain the specimen temperature at that of the human body. The Peltier ceramic, which has a square shape, is securely bonded to the specimen testing chamber using epoxy resin. The control board is mounted adjacent to the Arduino control board for the linear actuators.

### Specimen gripping system and force sensing

**Fig. 7:**
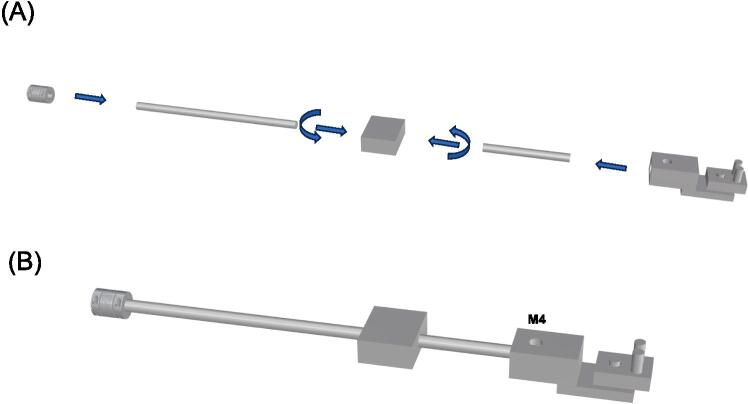
(A) Rods needs to be screw to the load cells and then the specimen grip can be mounted; (B) Fasten the specimen grip with M4 screws to have the final assembly of the load cell.

The gripping system comprises two main components: the grip base, which is connected to the rods using M6 screws, and the balanced pulley grip (namely, the grip) as shown by Fig. 7. These parts are assembled through a cylinder-to-hole connection to ensure the uniform distribution of applied loads during material testing, minimizing the risk of introducing non-physiological stress concentrations. To connect the rods to the actuators, rigid couplings are utilized, while the ends of the remaining rods are equipped with M8 screwdrivers for mounting the load cells. These load cells enable force measurements in orthogonal directions. The rods with the load cells are divided into two sections: i) the connection between the motor and load cell, and ii) the connection between the load cell and specimen grip. The load cell cables are connected to the amplifier and powered by a 12 V supply.

### Real-time displacement acquisition and graphical user interface

A semi-rigid arm is utilized to position the camera in close proximity to the sample area, enabling continuous marker tracking throughout the entire duration of the test. The camera is connected to the LabView software using a USB port. Once the CNC shield is mounted on the Arduino board, the non-captive linear motor can be connected to the stepper driver. The CNC shield is originally designed to manage three motors in the cartesian space (X, Y, Z). For the purpose of our machine, the linear motors aligned along the same directions of the biaxial cruciform configuration are connected in series. Thus, two linear motors are connected to the X direction of the CNC shield, while the perpendicular motors are connected to the Y direction of the control boards. To ensure optimal performance, a 30x30 mm fan is employed to cool the drivers. The driver control board is housed in a 3D-printed enclosure and powered by a 24 V supply.

## Operation instructions

**Fig. 8:**
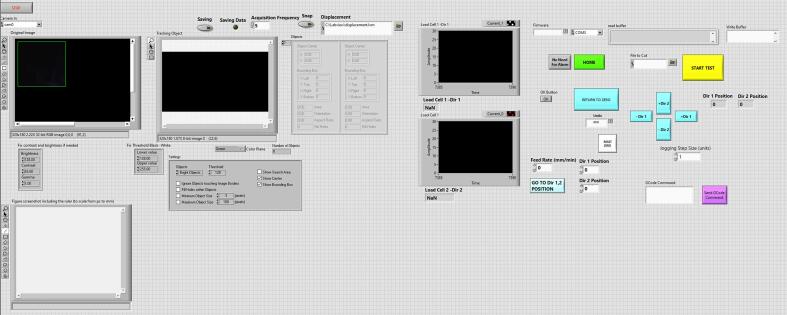
LabView front panel of input command and signal analysis of biaxial system.

The GUI program features four buttons on the front panel to facilitate motor movement in each direction (Fig. 8). The 'reset to zero' button allows for resetting the current motor positions, which is useful to establish a reference point before conducting material testing. The 'home' button enables the motors to return to the zero position after each specimen test whilst the 'start test' button is used to upload a set of G-code commands, simulating preconditioning followed by displacement-controlled biaxial stretching. Prior to testing, users can set the acquisition time frequency in seconds and provide a test name to save the data during the testing process. The load cell signals can be visualized as graphs or numeric values. The output data includes the X-Y coordinates of each marker (in pixels), motor displacements along each direction (in mm) and the load cell force signals (in N). After testing, an executable application implemented in Matlab (R2021a, Matworks, USA) allows for quick analysis of the output data and computation of stress and strain in both directions.

## Validation and characterization

The performance of the biaxial system is assessed through various tests and experiments conducted on springs and porcine biaxial samples. The accuracy of each linear actuator's motion is evaluated by comparing the target positions set in the GUI program with the displacement measurements obtained using a digital caliper. Three tests are performed for each motor, and the differences between the input and measured values are analyzed. This information is used to optimize the Grbl firmware settings by adjusting the step per mm for each linear actuator. The camera-based marker tracking tool is validated by acquiring and analyzing images of a grid composed of black dots with a diameter of 2.3 mm and a pitch of 7 mm. The LabView program is used to track and analyze the positions of these markers.

The analysis of the linear actuator performances reveals excellent agreement between the input commands and the actual motor positions. The relative errors (RE) in displacement measurements for different target displacements (1.0 mm, 5.0 mm, 10.0 mm, and 20.0 mm) are very low, ranging from 0.05% to 0.08%. This indicates that the linear actuators can accurately reach the desired displacements.

Similarly, the performance analysis of the marker tracking tool shows high agreement in the relative distances between markers in both the X and Y directions. The relative errors in distance measurements for the top and bottom markers in the X direction and the left and right markers in the Y direction are all below 0.07%. This suggests that the centroid estimations from the LabView program are stable and accurate.

To validate the response of the biaxial system, a stainless steel spring with certified mechanical properties is utilized. The minimum reaction load and the linear relationship between force and displacement in the spring are tested. The spring has an initial tension of 1.39 N, which represents the minimum force required to separate all its coils. By using the spring constant of 0.23 N/mm, the expected force can be estimated within the range of the actual displacements recorded during the uniaxial experimental testing of the spring. The results from testing the spring in both planar directions are then compared to the analytical solution as obtained from the product of spring constant and the displacement value.

**Fig. 9:**
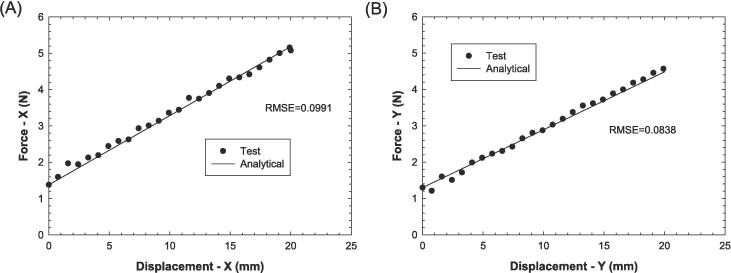
Comparison between experimental and analytical solution of spring material test for (A) X-direction and (B) Y-direction.

The force versus displacement curves obtained from the testing of the calibration spring also demonstrate good agreement between the experimental data and the analytical solution in both the X and Y directions (Fig. 9). The root mean square error (RMSE) values for the comparison of the experimental curves with the analytical solution are 0.079 and 0.0975 in the X and Y directions, respectively. This indicates that the biaxial system is capable of accurately quantifying the mechanical response of the calibration spring over a wide range of displacements.

**Fig. 10:**
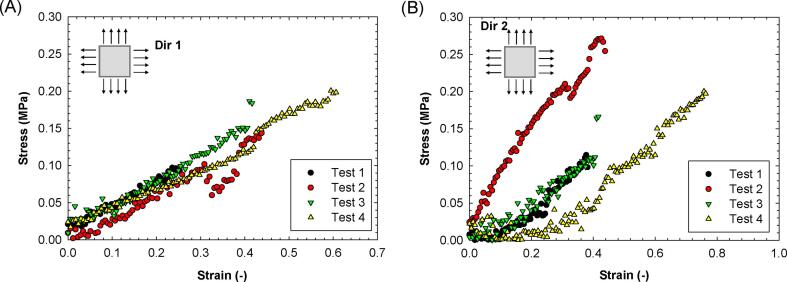
Biaxial stress–strain response of porcine aortic samples along (A) longitudinal (Dir 1) and (B) circumferential (Dir 2) vessel direction.

Finally, biaxial tests are conducted on several frozen porcine aorta using a previously published protocol to further evaluate and validate the performance of the biaxial system. In brief, square porcine samples (10x10 mm) were cur from the animal tissues and then steel racks are used for mounting the sample on the plan biaxial system. Testing was conducted under displacement-control conditions, with each grip subjected to a constant velocity of 1 mm/s following material failure. Before each test, the sample was pre-conditioned at 7% strain rate for ten cycles. Force and displacement were obtained to compute the engineering stress and engineering strain. Fig. 10 presents the stress–strain curves for the porcine aorta specimens, while Video 1 shows the biaxial test performed for a representative specimen. The porcine aorta samples exhibit a non-linear, hyperplastic material response, which is typical in biaxial testing investigations. The stress initially increases linearly with strain up to approximately 20%, followed by a steep increase in stress for higher strain magnitudes. The obtained stress–strain response of the porcine aorta samples was consistent with existing literature findings [20], [21], [22]. It's important to note that differences in the biomechanical response of the tissue could arise from factors such as the use of frozen tissue specimens instead of fresh ones [23], variations in the anatomic locations of the porcine aorta [24], and the inherent heterogeneity of material properties across different regions of the tissue [25].

The custom-built biaxial system presented in this study offers flexibility for further improvement and customization according to specific user needs. For instance, advanced gripping systems like can be developed using 3D printing manufacturing and easily mounted on the proposed testing system. The system here presented enables the material testing under controlled displacements since the feedback control of linear actuators cannot be implemented with the proposed hardware solution. However, from a software perspective, the real-time analysis of marker position could be utilized to adjust the motor position to ultimately maintain a constant marker position. Though the implementation of this approach is challenging, this may enables the development of creep and relaxation material testing protocols. Finally, our compact system can be easily integrated with fluorescence microscopy for live imaging and cell mechanics by replacing the middle of the cruciform testing chamber with a glass. We therefore conclude that our custom biaxial system offers an affordable and customizable solution for studying the mechanical behavior of soft tissues.

## Ethics statements

The animal study protocol was approved by the Institutional Review Board (or Ethics Committee) of University of Palermo (protocol code 04/04 and date of approval 01.01.2023).

## CRediT authorship contribution statement

**Salvatore Pasta:** Conceptualization, Writing – review & editing. **Chiara Catalano:** Methodology, Software, Writing – original draft. **Fabrizio Crascì:** Methodology, Software, Data curation. **Roberta Scuoppo:** Methodology, Software, Validation.

## Declaration of Competing Interest

The authors declare that they have no known competing financial interests or personal relationships that could have appeared to influence the work reported in this paper.

## References

[1] Pasta S., Agnese V., Gallo A., Cosentino F., Di Giuseppe M., Gentile G., Raffa G.M., Maalouf J.F., Michelena H.I., Bellavia D., Conaldi P.G., Pilato M. (2020). Shear stress and aortic strain associations with biomarkers of ascending thoracic aortic aneurysm. Ann. Thorac. Surg..

[2] Pasta S., Gentile G., Raffa G.M., Scardulla F., Bellavia D., Luca A., Pilato M., Scardulla C. (2017). Three-dimensional parametric modeling of bicuspid aortopathy and comparison with computational flow predictions. Artif. Organs.

[3] Scardulla F., Pasta S., D’Acquisto L., Sciacca S., Agnese V., Vergara C., Quarteroni A., Clemenza F., Bellavia D., Pilato M. (2017). Shear stress alterations in the celiac trunk of patients with a continuous-flow left ventricular assist device as shown by in-silico and in-vitro flow analyses. J. Heart Lung Transplant.

[4] Duprey A., Trabelsi O., Vola M., Favre J.-P., Avril S. (2016). Biaxial rupture properties of ascending thoracic aortic aneurysms. Acta Biomater..

[5] May-Newman K., Yin F.C. (1995). Biaxial mechanical behavior of excised porcine mitral valve leaflets. Am. J. Phys. Anthropol..

[6] Sacks M.S. (2000). Biaxial mechanical evaluation of planar biological materials. J. Elast..

[7] Pichamuthu J.E., Phillippi J.A., Cleary D.A., Chew D.W., Hempel J., Vorp D.A., Gleason T.G. (2013). Differential tensile strength and collagen composition in ascending aortic aneurysms by aortic valve phenotype. Ann. Thorac. Surg..

[8] Di Giuseppe M., Alotta G., Agnese V., Bellavia D., Raffa G.M., Vetri V., Zingales M., Pasta S., Pilato M. (2019). Identification of circumferential regional heterogeneity of ascending thoracic aneurysmal aorta by biaxial mechanical testing. J. Mol. Cell. Cardiol..

[9] Pasta S., Phillippi J.A., Gleason T.G., Vorp D.A. (2012). Effect of aneurysm on the mechanical dissection properties of the human ascending thoracic aorta. J. Thorac. Cardiovasc. Surg..

[10] Duprey A., Khanafer K., Schlicht M., Avril S., Williams D., Berguer R. (2010). In vitro characterisation of physiological and maximum elastic modulus of ascending thoracic aortic aneurysms using uniaxial tensile testing. Eur. J. Vasc. Endovasc. Surg..

[11] Hemmasizadeh A., Autieri M., Darvish K. (2012). Multilayer material properties of aorta determined from nanoindentation tests. J. Mech. Behav. Biomed. Mater..

[12] Bae Y.H. (2016). Measuring the stiffness of ex vivo mouse aortas using atomic force microscopy. Jove-J. Visual. Experiments.

[13] Holzapfel G.A., Ogden R.W. (1902). Constitutive modelling of passive myocardium: a structurally based framework for material characterization. Philos. Trans. A Math. Phys. Eng. Sci..

[14] Romo A., Badel P., Duprey A., Favre J.-P., Avril S. (2014). In vitro analysis of localized aneurysm rupture. J. Biomech..

[15] Shiwarski D.J., Tashman J.W., Eaton A.F., Apodaca G., Feinberg A.W. (2020). 3D printed biaxial stretcher compatible with live fluorescence microscopy. Hardwarex.

[16] Cavinato C., Murtada S.-I., Rojas A., Humphrey J.D. (2021). Evolving structure-function relations during aortic maturation and aging revealed by multiphoton microscopy. Mech. Ageing Dev..

[17] D'Ancona G., Amaducci A., Rinaudo A., Pasta S., Follis F., Pilato M., Baglini R. (2013). Haemodynamic predictors of a penetrating atherosclerotic ulcer rupture using fluid-structure interaction analysis. Interact. Cardiovasc. Thorac. Surg..

[18] Pasta S., Phillippi J.A., Tsamis A., D'Amore A., Raffa G.M., Pilato M., Scardulla C., Watkins S.C., Wagner W.R., Gleason T.G., Vorp D.A. (2016). Constitutive modeling of ascending thoracic aortic aneurysms using microstructural parameters. Med. Eng. Phys..

[19] Fratini L., Pasta S. (2012). Residual stresses in friction stir welded parts of complex geometry. Int. J. Adv. Manuf. Technol..

[20] Deplano V., Boufi M., Boiron O., Guivier-Curien C., Alimi Y., Bertrand E. (2016). Biaxial tensile tests of the porcine ascending aorta. J. Biomech..

[21] Nicosia M.A. (2002). Biaxial mechanical properties of porcine ascending aortic wall tissue. J. Heart Valve Dis..

[22] Zeinali-Davarani, S., et al., Identification of in vivo material and geometric parameters of a human aorta: toward patient-specific modeling of abdominal aortic aneurysm. Biomech Model Mechanobiol. 10.1007/s10237-010-0266-y 21053043

[23] Deveja R.P., Iliopoulos D.C., Kritharis E.P., Angouras D.C., Sfyris D., Papadodima S.A., Sokolis D.P. (2018). Effect of aneurysm and bicuspid aortic valve on layer-specific ascending aorta mechanics. Ann. Thorac. Surg..

[24] Iliopoulos D.C., Deveja R.P., Kritharis E.P., Perrea D., Sionis G.D., Toutouzas K., Stefanadis C., Sokolis D.P. (2009). Regional and directional variations in the mechanical properties of ascending thoracic aortic aneurysms. Med. Eng. Phys..

[25] Sassani S.G., Tsangaris S., Sokolis D.P. (2015). Layer- and region-specific material characterization of ascending thoracic aortic aneurysms by microstructure-based models. J. Biomech..

